# Distinct Longitudinal Trajectories of SLEDAI‐2K Scores Predict Prognosis in Systemic Lupus Erythematosus Based on Group‐Based Trajectory Modeling

**DOI:** 10.1155/jimr/5322286

**Published:** 2026-06-30

**Authors:** Renren Ouyang, Ali Xiong, Xiaochun Shu, Ting Wang, Wei Wei, Rujia Chen, Feng Wang, Hongyan Hou, Shiji Wu

**Affiliations:** ^1^ Department of Laboratory Medicine, Tongji Hospital of Tongji Medical College of Huazhong University of Science and Technology, Wuhan, Hubei, China, hust.edu.cn; ^2^ Department of Laboratory Medicine, The Central Hospital of Wuhan, Tongji Medical College, Huazhong University of Science and Technology, Wuhan, China, hust.edu.cn

**Keywords:** disease activity, group-based trajectory modeling, six month clinical status, SLEDAI-2K, systemic lupus erythematosus

## Abstract

**Objective:**

To apply group‐based trajectory modeling (GBTM) to longitudinal real world data from patients with systemic lupus erythematosus (SLE) to identify distinct disease activity trajectories and factors associated with 6 month clinical status.

**Methods:**

A total of 91 patients with SLE treated between 2017 and 2024 were included. SLE Disease Activity Index 2000 (SLEDAI‐2K) scores were assessed at baseline, 3 months, and 6 months. GBTM was used to characterize longitudinal disease activity patterns. Heatmap clustering analysis was performed to compare clinical laboratory variables and SLEDAI‐2K scores across trajectory groups and to further explore their association with 6 month clinical status.

**Results:**

Two distinct disease activity trajectories were identified. Class 1, comprising patients with higher initial SLEDAI‐2K scores, was predominantly composed of nonremission cases (90.91%). Class 2, characterized by lower initial SLEDAI‐2K scores, showed a more balanced distribution of nonremission (46.81%) and remission (53.19%) patients. At 6 months, the remission subgroup had significantly lower SLEDAI‐2K scores and lower urinary biomarker levels, including 24 h urinary microalbumin (24 h‐UMA), 24 h urinary micrototal protein (24 h‐UMTP), UMA, and UMTP, than the nonremission subgroup.

**Conclusions:**

Longitudinal monitoring of SLEDAI‐2K trajectories may help identify patients at risk for persistent high disease activity or nonremission at 6 months and those who may benefit from closer monitoring and treatment adjustment. These findings support a stratified management approach in SLE based on trajectory profiles.

**Summary**

• Systemic lupus erythematosus (SLE) is a heterogeneous autoimmune disease, and previous studies have predominantly used cross‐sectional designs to assess disease activity.
• This study applied group‐based trajectory modeling (GBTM) to identify distinct longitudinal patterns of disease activity in SLE patients based on SLE Disease Activity Index 2000 (SLEDAI‐2K) scores.
• Urinary biomarkers, such as 24 h urinary microalbumin (24 h‐UMA), 24 h urinary micrototal protein (24 h‐UMTP), and UMA, were associated with short‐term disease activity patterns and treatment response.
• The identification of distinct disease trajectories may support early clinical stratification and inform personalized management strategies in SLE.

## 1. Introduction

Systemic lupus erythematosus (SLE) is a chronic autoimmune disease characterized by multisystem involvement and a relapsing remitting clinical course [1]. Its clinical manifestations are highly heterogeneous, ranging from constitutional and mucocutaneous features, such as fever, alopecia, cutaneous eruptions, and oral ulcers, to arthritis, hematologic abnormalities, serositis, lupus nephritis with proteinuria, and neuropsychiatric involvement [2]. Laboratory abnormalities commonly include normocytic anemia, thrombocytopenia, leukopenia, hypocomplementemia, serum and urinary biomarkers suggestive of lupus nephritis, elevated serum creatinine, and immunologic abnormalities such as antinuclear antibodies (ANAs) and anti‐double‐stranded DNA (anti‐dsDNA) antibodies [3]. Owing to this marked heterogeneity in both clinical and laboratory manifestations, accurate diagnosis, and disease monitoring remain challenging in routine practice [4, 5].

Accurate assessment of disease activity is essential for guiding therapy and evaluating the clinical course in patients with SLE. The SLE Disease Activity Index (SLEDAI) is a widely used instrument for measuring disease activity in SLE [6]. SLEDAI‐2K is a revised version of the original SLEDAI that allows persistent active manifestations present over the preceding 30 days, such as rash, alopecia, mucosal ulcers, and proteinuria, to be captured at follow‐up, thereby making it more suitable for longitudinal assessment. However, despite its clinical usefulness, SLEDAI‐2K still primarily provides cross‐sectional snapshots and has limited sensitivity for detecting partial improvement or deterioration because of its dichotomous scoring system. Each item is scored as either present or absent without accounting for severity or incremental changes in manifestations [7]. This limitation restricts the ability to reflect nuanced fluctuations in disease activity over time. Consequently, while SLEDAI‐2K remains a valuable tool for identifying active diseases, its usefulness in monitoring incremental changes and guiding treatment adjustments remains limited.

Traditional approaches, such as time‐adjusted summary measures including the adjusted mean SLEDAI (AMS), have been used to quantify cumulative disease activity over defined periods. AMS is calculated by integrating serial SLEDAI values across follow‐up and dividing by the observation time, thereby providing a time‐weighted average of disease activity. However, such summary measures may not fully capture the dynamic and heterogeneous nature of SLE progression [8]. A study based on the Hopkins Lupus Cohort proposed a random effects model in which yearly mean disease activity was estimated as a function of the previous year’s mean disease activity together with other covariates; this model was later validated in the Systemic Lupus International Collaborating Clinics (SLICC) inception cohort [9]. These findings suggest that higher disease activity may reduce subsequent variability. Building on these observations, recent studies have highlighted the importance of longitudinal evaluation of disease activity trajectories in SLE. Group‐based trajectory modeling (GBTM) has emerged as a useful statistical approach for identifying distinct subpopulations within longitudinal datasets and for characterizing heterogeneous patterns of disease evolution and treatment response [10]. For example, a previous study using GBTM identified four distinct anxiety trajectories among patients with SLE and showed that anxiety levels remained relatively stable over time regardless of fluctuations in disease activity, further supporting the usefulness of this method in capturing disease heterogeneity [11].

Because renal involvement is a major determinant of clinical outcomes in SLE, considerable attention has been directed toward urinary biomarkers that reflect renal injury and disease activity. Traditional markers, such as proteinuria and serum creatinine, are widely used but have limited sensitivity and specificity [12]. Emerging urinary biomarkers, including monocyte chemoattractant protein‐1 (MCP‐1), neutrophil gelatinase‐associated lipocalin (NGAL), and kidney injury molecule‐1 (KIM‐1), have shown promise in detecting renal inflammation and predicting flares in lupus nephritis [13–15]. Integrating urinary biomarkers into longitudinal assessments of disease activity may improve the characterization of disease trajectories and facilitate the earlier identification of patients at risk for persistent disease activity. Therefore, in this study, we integrated urinary biomarker profiles with SLEDAI‐2K scores to characterize distinct disease activity trajectories and explore their clinical relevance in a real‐world SLE cohort.

## 2. Methods

### 2.1. Subjects

A total of 91 patients with newly diagnosed SLE were included in this historical cohort study at Tongji Hospital, Tongji Medical College, Huazhong University of Science and Technology between 2017 and 2024. Clinical data were obtained from the existing medical records, and no randomization was performed. To ensure consistency in case definition across the study period, all included patients were reevaluated according to the 2019 European League Against Rheumatism/American College of Rheumatology (EULAR/ACR) classification criteria during the present analysis, and no change in case eligibility was observed after reclassification. Only patients with complete follow‐up assessments at baseline, month 3, and month 6 were included in the final analysis.

Disease activity was assessed using SLEDAI‐2K. SLEDAI‐2K is a validated global disease activity index that quantifies disease activity over the preceding 10 days using 24 weighted clinical and laboratory descriptors, including neurologic, renal, musculoskeletal, serosal, mucocutaneous, immunologic, constitutional, and hematologic manifestations. Each descriptor is scored as present or absent according to predefined criteria, and the total score reflects the overall disease burden (Supporting Information 1: Table S1). At each evaluation time point, clinical manifestations and laboratory parameters, including complete blood count, urinalysis, complement levels (C3 and C4), and anti‐dsDNA antibody titers, were reviewed and scored according to the standardized SLEDAI‐2K criteria. After systemic treatment, patients with a SLEDAI‐2K score >4 were classified as having high disease activity (nonremission), whereas those with a score ≤4 were classified as having low disease activity (remission). All assessments were performed by trained clinicians to ensure consistency, and any discrepancies were resolved through consensus discussion among rheumatologists experienced in SLE management.

Strict exclusion criteria were applied. Patients with active infections were excluded based on clinical evaluation, laboratory investigations, and radiologic examinations when clinically indicated. Malignancies were excluded through comprehensive clinical assessment and auxiliary investigations, as needed. Individuals with coexisting rheumatic diseases were excluded following detailed clinical evaluation by experienced rheumatologists, supported by disease‐specific autoantibody testing and established diagnostic criteria. All participants provided written informed consent. This study was approved by the Ethics Committee of Tongji Hospital, Tongji Medical College, Huazhong University of Science and Technology (TJ‐IRB202308129).

### 2.2. Monitoring of Disease Activity and Other Clinical Parameters

Demographic data, medical history, and laboratory results were extracted from the hospital electronic medical record system. All laboratory investigations were performed as part of routine clinical care in the central laboratory of Tongji Hospital using standardized operating procedures and quality‐controlled platforms throughout the study period. Laboratory assessments included ANAs, anti‐dsDNA (anti‐dsDNA) antibodies, complete blood counts, liver and kidney function tests, and urinalysis. ANA profiles were measured using the BioPlex 2200 System (Bio‐Rad Laboratories), which enables the simultaneous detection of multiple autoantibodies. Anti‐dsDNA antibodies were detected by an indirect immunofluorescence assay (IIFA) using the EUROPattern computer‐aided immunofluorescence microscopy system (EUROIMMUN). Complete blood counts were performed using a Sysmex automated hematology analyzer. Liver and kidney function parameters, including alanine aminotransferase (ALT), aspartate aminotransferase (AST), serum creatinine, and blood urea nitrogen (BUN), were measured using routine automated biochemical laboratory platforms.

### 2.3. Lymphocyte Subset Analysis by Flow Cytometry

The percentages and absolute numbers of CD4^+^ T cells, CD8^+^ T cells, B cells, and NK cells were determined using TruCOUNT tubes and the BD Multitest 6‐Color TBNK Reagent Kit (BD Biosciences, San Jose, CA, USA) according to the manufacturer’s instructions. Briefly, 50 μL of whole blood was incubated with the six‐color antibody cocktail for 15 min at room temperature, followed by the addition of 450 μL of FACS lysing solution. Samples were then analyzed using a BD FACSCanto flow cytometer equipped with FACSCanto clinical software. Data acquisition and analysis were performed according to standardized protocols. The full gating strategy for flow cytometry analysis is shown in Supporting Information 2: Figure S1.

### 2.4. Statistical Analysis and GBTM

Quantitative variables were summarized as medians and interquartile ranges (IQRs). Comparisons between groups were performed using the Chi‐square test or Fisher’s exact test for categorical variables, as appropriate, and the Mann–Whitney *U* test or Kruskal–Wallis test for continuous variables, as appropriate. GBTM was performed using the “trajeR” package in R (version 4.4.1) to identify clusters of patients with similar disease activity patterns over time. The optimal number of trajectory groups was determined primarily based on the lowest adjusted Bayesian information criterion (aBIC) while also considering entropy, mean posterior probability of class membership, class size, and clinical interpretability.

Models with two to four latent classes were initially evaluated. The final model was selected on the basis of model fit, classification adequacy, subgroup size, and clinical interpretability. After the trajectory groups were identified, clinical outcomes were further assessed within each group. For patients in Class 2, remission and nonremission subgroups were compared with respect to baseline characteristics, dynamic clinical and laboratory changes at month 3 and month 6, and treatment patterns over time. Heatmap clustering analysis was subsequently used to visualize the overall distribution patterns of key clinical variables. All statistical analyses were performed in R, and a two‐tailed *p*‐value <0.05 was considered statistically significant.

## 3. Results

### 3.1. Baseline Characteristics of the Study Population

A total of 91 patients with newly diagnosed SLE were included in the study. Among them, 29 (31.87%) were classified as the remission group and 62 (68.13%) as the nonremission group. Baseline demographic and clinical characteristics are summarized in Table 1. The median ages were 37 years in the remission group and 43 years in the nonremission group, and the majority of patients in both groups were female. At baseline, patients in the remission group had significantly lower SLEDAI‐2K scores than those in the nonremission group. Urinary biomarkers, including 24 h urinary microalbumin (24 h‐UMA), 24 h urinary micrototal protein (24 h‐UMTP), and UMA, were also significantly lower in the remission group (*p* < 0.05). In addition, the proportion of total B cells was lower in the remission group than in the nonremission group. No significant differences were observed in routine blood parameters, liver function, renal function, or coagulation markers between the two groups.

**Table 1:** The clinical and laboratory characteristics of patients in remission and nonremission groups.

Parameters	Remission group (*n* = 29)	Nonremission group (*n* = 62)	*p*‐Value
Age, median [IQR], year	37.000 [16.000–55.000]	43.000 [25.000–59.000]	0.241
Sex			
Male, *n* (%)	6 (20.690)	7 (11.290)	—
Female, *n* (%)	23 (79.310)	55 (88.710)	0.232
SLEDAI‐2K score	9.000 [8.000,12.000]	12.000 [8.000,16.000]	0.007
Blood routine indictors			
WBC (×10^9^/L)	4.530 [3.590–6.300]	4.900 [3.860–6.960]	0.509
Lymphocytes (×10^9^/L)	0.870 [0.750–1.070]	0.940 [0.660–1.600]	0.528
Neutrophils (×10^9^/L)	3.170 [2.140–4.920]	3.440 [2.220–4.910]	0.609
RBC (×10^12^/L)	3.416 ± 0.750	3.297 ± 0.812	0.509
Hemoglobin (g/L)	98.310 ± 21.411	95.677 ± 23.420	0.613
Platelet (×10^9^/L)	155.000 [107.000–222.000]	149.000 [112.000–210.000]	0.956
Blood biochemistry indicators			
Total protein (g/L)	62.628 ± 12.235	58.627 ± 12.449	0.159
Globulin (g/L)	33.000 [27.700–38.200]	28.900 [24.300–35.600]	0.392
eGFR (mL/min/1.73 m^2^)	79.756 ± 31.441	72.977 ± 35.227	0.483
ALP (U/L)	69.000 [54.000–91.000]	56.000 [45.000–76.000]	0.011
GGT (U/L)	27.000 [14.000–37.000]	21.000 [14.000–31.000]	0.167
Cr (μmol/L)	72.000 [51.000–103.000]	85.000 [62.000–133.000]	0.137
Coagulation markers			
PT (s)	12.900 [12.000–13.500]	12.800 [12.400–13.500]	0.24
FIB (g/L)	3.820 [2.840–4.480]	4.000 [3.190–4.670]	0.345
APTT (s)	3.820 [2.840–4.480]	4.000 [3.190–4.670]	0.345
TT (s)	17.300 [16.800–18.500]	17.200 [16.500–17.800]	0.764
D‐dimer (μg/mL)	1.780 [0.840–4.000]	1.630 [0.830–2.620]	0.648
Urinary indictors			
U‐RBC (/μL)	55.600 [25.100–109.000]	101.100 [25.400–426.500]	0.061
U‐WBC (/μL)	35.400 [10.000–45.900]	45.000 [21.200–116.000]	0.077
Urinary casts (/μL)	0.300 [0.000–0.500]	0.300 [0.000–1.900]	0.645
24 h‐UMA (mg/24 h)	879.800 [204.200–2415.400]	1533.000 [912.700–4286.100]	0.04
24 h‐UMTP (mg/24 h)	1050.300 [420.400–3315.000]	2199.600 [1340.500–5000.300]	0.022
UMA (mg/L)	743.200 [424.700–2550.500]	1776.300 [702.100–4400.000]	0.024
UMTP (mg/L)	1147.000 [647.000–3515.000]	2488.000 [1233.000–6144.000]	0.065
Immunity indictors			
Total T cell (%)	78.798 ± 8.964	73.559 ± 11.451	0.053
Total B cells (%)	12.861 ± 6.854	20.094 ± 12.302	0.002
NK (%)	4.980 [2.980–8.240]	4.720 [2.300–6.900]	0.702
TBNK (%)	99.150 [98.800–99.500]	99.250 [99.000–99.530]	0.283
CD4 T cells (%)	33.571 ± 11.059	33.389 ± 9.836	0.943
CD8 T cells (%)	37.350 [30.860–48.220]	32.970 [27.930–43.780]	0.151
C3 (g/L)	0.480 [0.200–0.600]	0.380 [0.280–0.480]	0.356
C4 (g/L)	0.090 [0.040–0.160]	0.060 [0.040–0.130]	0.482
IgA (g/L)	2.600 [1.790–3.510]	2.210 [1.730–3.280]	0.612
IgG (g/L)	2.600 [1.790–3.510]	2.210 [1.730–3.280]	0.612
IgM (g/L)	1.160 [0.710–1.790]	0.930 [0.710–1.330]	0.19

*Note*: Data are presented as *n* (%), median (IQR), or mean ± SD.

Abbreviations: 24 h‐UMA, 24 hour urinary microalbumin; 24 h‐UMTP, 24 hour urinary micrototal protein; ALP, alkaline phosphatase; APTT, activated partial thromboplastin time; C3, complement C3; C4, complement C4; Cr, creatinine; FIB, fibrinogen; GGT, gamma glutamyl transferase; IgA, immunoglobulin A; IgG, immunoglobulin G; IgM, immunoglobulin M; PT, prothrombin time; RBC, red blood cell count; TBNK, total T, B, and NK lymphocyte subsets; TT, thrombin time; UMA, urinary microalbumin; UMTP, urinary micrototal protein; U‐RBC, urinary red blood cell count; U‐WBC, urinary white blood cell count; WBC, white blood cell count.

### 3.2. Longitudinal Changes in Disease Activity and Laboratory Indicators

**Figure 1:**
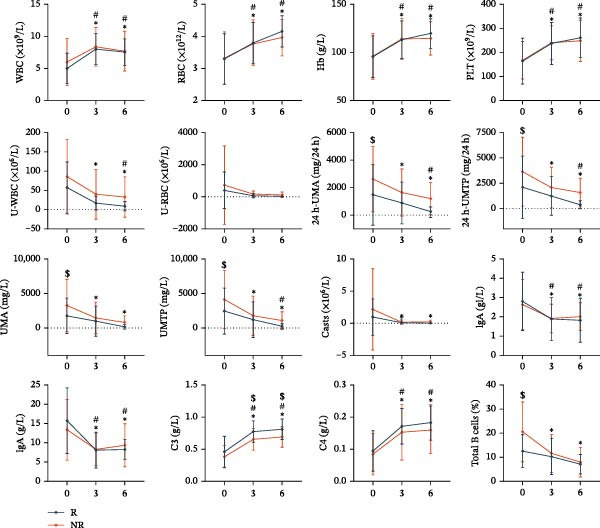
Dynamic changes in laboratory parameters in the remission (R, *n* = 29) and nonremission (NR, *n* = 62) groups at baseline, month 3, and month 6. Parameters shown include WBC, RBC, Hb, PLT, U‐WBC, U‐RBC, 24 h‐UMA, 24 h‐UMTP, UMA, UMTP, urinary casts, IgA, IgG, C3, C4, and total B cells. Comparisons within groups across time points and between groups at the same time point were performed as indicated in the figure.  ^∗^ *p* < 0.05 versus baseline within the NR group; ^#^ *p* < 0.05 versus baseline within the R group; ^$^ *p* < 0.05 between groups at the same time point.

**Figure 2:**
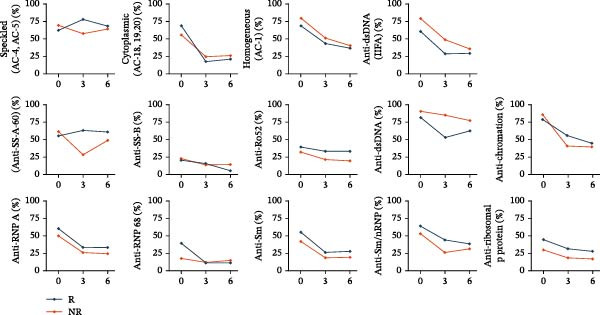
Dynamic changes in autoantibody profiles in the remission (R, *n* = 29) and nonremission (NR, *n* = 62) groups at baseline, month 3, and month 6. Autoantibody related variables shown include ANA patterns, including speckled (AC‐4 and AC‐5), cytoplasmic (AC‐18, 19, and 20), and homogeneous (AC‐1), together with serum autoantibodies detected by the BioPlex 2200 system, including anti‐dsDNA, anti‐SSA‐60, anti‐SS‐B, anti‐Ro52, anti‐chromatin, anti‐RNP A, anti‐RNP 68, anti‐Sm, anti‐Sm/RNP, and anti‐ribosomal P protein.

Following systemic treatment, both groups showed a gradual decline in disease activity over the 6‐month follow‐up period, as illustrated in Figures 1 and 2. In the remission group, complement C3 and C4 levels increased significantly over time, accompanied by marked decreases in ANA and anti‐dsDNA titers compared with the nonremission group. In addition, although urinary white blood cells (U‐WBC), 24 h‐UMA, 24 h‐UMTP, UMTP, serum IgA, and IgG levels showed decreasing trends in both groups, these reductions were more pronounced in the remission group. The remission group also showed a significant reduction in urinary casts and a downward trend in total B cell percentages, whereas these changes were not statistically significant in the nonremission group. Regarding serological autoantibodies, a general downward trend in all nuclear antigen indicators was observed over time (Figure 2), suggesting improvement in immunological status after treatment.

### 3.3. GBTM of SLEDAI‐2K Scores

GBTM was performed to classify patients according to longitudinal SLEDAI‐2K score patterns. The optimal model fit was determined on the basis of the lowest aBIC values (Supporting Information 3: Table S2). Two distinct trajectories were identified. Class 1, comprising 48.35% of patients, was characterized by higher initial SLEDAI‐2K scores and a gradual decline in disease activity over the follow‐up period. The majority of patients in this class (90.91%) remained in the nonremission group. In contrast, Class 2, representing 51.65% of patients, showed lower initial SLEDAI‐2K scores and relatively stable disease activity. Within Class 2, 53.19% of patients achieved remission, whereas 46.81% remained nonremission (Table 2).

**Table 2:** Clinical and laboratory characteristics of patients in class 1 and class 2.

Parameters	Class 1 (*n* = 44)	Class 2 Group (*n* = 47)	*p*‐Value
Age, median [IQR], years	43.000 [26.000–59.000]	38.000 [18.000–54.000]	0.241
Sex			
Male, *n* (%)	6 (13.636)	7 (14.894)	—
Female, *n* (%)	38 (86.364)	40 (85.106)	0.864
SLEDAI‐2K score	15.000 [11.000–17.000]	9.000 [8.000–11.000]	<0.001
Urinary indicators			
U‐RBC (/μL)	117.800 [47.300–452.200]	55.700 [22.300–151.900]	0.052
U‐WBC (/μL)	75.500 [36.000–121.700]	22.400 [12.700–41.200]	<0.001
Urinary casts (/μL)	0.400 [0.000–2.000]	0.200 [0.000–0.400]	0.047
24 h‐UMA (mg/24 h)	1746.400 [1038.100–4629.500]	879.800 [204.200–2415.400]	0.007
24 h‐UMTP (mg/24 h)	2273.800 [1450.800–5559.000]	1315.600 [445.400–3177.900]	0.004
UMA (mg/L)	2277.300 [936.800–5012.800]	923.300 [424.700–3128.100]	0.007
UMTP (mg/L)	3164.000 [1511.000–6368.000]	1405.000 [644.000–3515.000]	0.007
Immune indicators			
Total T cell (%)	74.580 [68.860–79.430]	78.340 [70.710–85.680]	0.103
Total B cells (%)	20.540 [12.000–26.790]	12.260 [7.180–20.320]	0.024
TBNK (%)	99.370 [99.110–99.630]	99.030 [98.660–99.450]	0.021
C3 (g/L)	0.342 ± 0.146	0.477 ± 0.223	0.001
C4 (g/L)	0.050 [0.030–0.110]	0.090 [0.050–0.150]	0.066

*Note*: Data are presented as *n* (%), median (interquartile range), or mean ± standard deviation (SD).

Abbreviations: 24 h‐UMA, 24‐hour urinary microalbumin; 24 h‐UMTP, 24‐hour urinary micrototal protein; C3, complement C3; C4, complement C4; TBNK, total T, B, and NK lymphocyte subsets; UMA, urinary microalbumin; UMTP, urinary micrototal protein; U‐RBC, urinary red blood cell count; U‐WBC, urinary white blood cell count.

Comparisons of baseline characteristics between Class 1 and Class 2 are summarized in Table 2. Patients in Class 1 had significantly higher U‐WBC counts, 24 h‐UMA, 24 h‐UMTP, UMA, and total B cell percentages, together with lower complement C3 levels, than patients in Class 2 (all *p* < 0.05). These findings indicate that higher baseline disease activity and renal involvement markers were associated with a less favorable disease activity trajectory.

### 3.4. Clinical Outcomes Within Trajectory Groups

**Figure 3:**
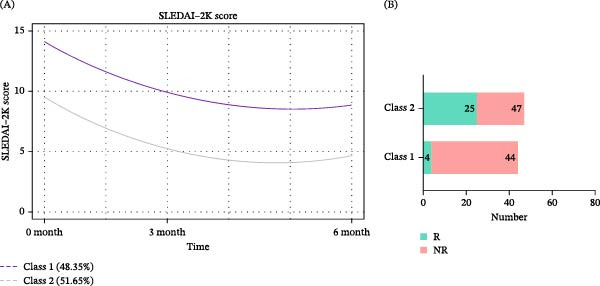
Group based trajectory modeling of SLEDAI‐2K scores in patients with SLE (*n* = 91). (A) Estimated longitudinal trajectories of SLEDAI‐2K scores from baseline to month 6. (B) Distribution of remission (R) and nonremission (NR) patients within each trajectory class. Class 1 included 44 patients and Class 2 included 47 patients.

**Figure 4:**
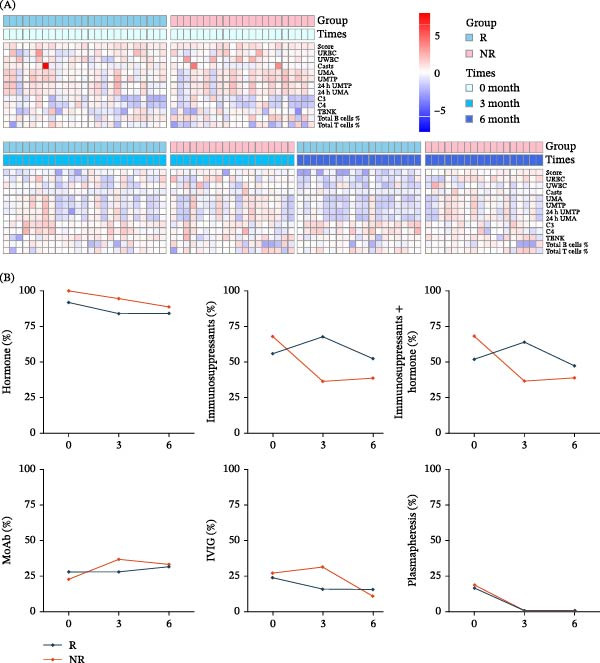
Clinical characteristics and treatment patterns in Class 2 stratified by remission status. (A) Heatmap clustering analysis of key clinical and laboratory variables in remission (R, *n* = 25) and nonremission (NR, *n* = 22) patients within Class 2 (*n* = 47) at baseline, month 3, and month 6. (B) Longitudinal changes in treatment patterns, including corticosteroid therapy, immunosuppressants, combined corticosteroid and immunosuppressant therapy, monoclonal antibody therapy, intravenous immunoglobulin, and plasmapheresis, in the R and NR groups within Class 2 at baseline, month 3, and month 6.

Analysis of clinical outcomes within trajectory groups showed that patients in Class 1 predominantly remained nonremission throughout the study period (Figure 3B). By contrast, patients in Class 2 were nearly evenly distributed between the remission and nonremission subgroups (53.19% vs. 46.81%), with no significant differences in baseline clinical and laboratory characteristics between these two subgroups (Supporting Information 4: Table S3). To further distinguish 6 months clinical status within Class 2, dynamic changes at 3 and 6 months were evaluated. Heatmap clustering analysis (Figure 4A) showed that at 6 months, remission patients in Class 2 had significantly lower SLEDAI‐2K scores and lower levels of U‐RBC, U‐WBC, 24 h‐UMA, 24 h‐UMTP, UMA, and UMTP than nonremission patients. In addition, medication use patterns differed between remission and nonremission subgroups. At baseline, a lower proportion of patients in the remission subgroup received corticosteroids and immunosuppressants; however, by 3 months, the proportion receiving immunosuppressive therapy had increased significantly and exceeded that in the nonremission subgroup (Figure 4B). These findings suggest that early adjustment of immunosuppressive regimens, particularly those targeting renal involvement, may be associated with disease remission in patients with SLE.

## 4. Discussion

SLE is a heterogeneous autoimmune disease with manifestations ranging from mild mucocutaneous involvement to severe organ‐threatening complications, posing substantial challenges for disease monitoring and treatment individualization [16, 17]. Because cross sectional assessments often fail to capture within patient fluctuations, longitudinal analytical approaches may provide additional clinically relevant information [18]. Using GBTM, we identified two distinct 6 month SLEDAI‐2K trajectories: a high baseline activity group with limited improvement that remained predominantly nonremission and a lower baseline activity group with a more favorable course and a balanced remission distribution. These patterns are consistent with previous studies highlighting the heterogeneity of disease activity courses in SLE [11].

Although our cohort was modest in size, candidate models with one to four latent classes were systematically compared, and the two class solution was selected using a multidimensional set of criteria, including aBIC, entropy, average posterior probabilities, class size, and clinical interpretability. The selected model showed the lowest aBIC, entropy greater than 0.80, posterior probabilities greater than 0.85, and avoided very small and clinically indistinct subgroups, supporting reliable separation of latent classes in a moderate real‐world dataset [10]. Importantly, remission at 6 months, defined as SLEDAI‐2K less than or equal to 4, was not included in the trajectory modeling process. Rather, it was used post hoc to evaluate whether the data‐driven classes corresponded to independently defined short‐term clinical status, thereby reducing the risk of circular interpretation.

Lupus nephritis remains a major determinant of clinical outcomes in SLE, affecting approximately 30%–60% of patients and contributing substantially to the risk of chronic kidney disease and progression to end‐stage kidney disease despite immunosuppressive therapy [19–22]. In our cohort, the high activity trajectory showed higher baseline urinary protein‐related indices, including 24 h‐UMA, 24 h‐UMTP, and UMA, together with lower C3 levels, findings consistent with renal involvement and previously associated with less favorable renal outcomes [22–24]. We recognize that these urinary protein‐related measures are nonspecific markers of proteinuria and kidney injury rather than direct indicators of inflammatory activity. Nevertheless, they are widely accessible in routine clinical practice and show dynamic changes over time in our dataset. Emerging urinary biomarkers, such as NGAL, MCP‐1, and proteomic panels, may offer greater specificity for active lupus nephritis and flare prediction and should be incorporated into future prospective studies to better define the biologic basis of trajectory profiles [12–15].

Medication use patterns were analyzed descriptively rather than incorporated as time‐varying covariates because treatment timing, intensity, and dose standardization were heterogeneous in this historical cohort. Notably, patients who achieved remission showed earlier escalation of immunosuppressive therapy by month 3, suggesting that proactive treatment intensification may be associated with a more favorable short‐term disease course. However, this observation should not be interpreted causally, and future studies with standardized treatment exposure data will be needed to formally evaluate the impact of therapy on trajectory transitions.

In the present study, the clinical implication of the identified trajectories was limited to short‐term clinical status at 6 months, specifically remission versus nonremission, rather than to intermediate or long‐term outcomes such as renal flare, hospitalization, damage accrual, or mortality. We have, therefore, moderated the corresponding wording throughout the manuscript. Although 6 months remission is clinically meaningful and may be associated with improved renal outcomes when proteinuria declines early [25, 26]. Extended follow‐up will be required to determine whether early trajectory membership predicts long‐term organ outcomes.

SLEDAI‐2K is a validated and widely used measure in both clinical trials and routine practice [6, 18]. However, because each item is scored dichotomously, the instrument may have limited sensitivity to partial improvement or mild relapse over short intervals, thereby attenuating subtle temporal changes [27–29]. Composite or more continuous disease activity measures, as well as multidomain responder definitions, may offer greater granularity for longitudinal profiling, but these were not uniformly available in the present dataset [28]. Future prospective studies incorporating multidimensional disease control definitions together with physician‐reported and patient‐reported measures may further refine disease trajectory characterization.

Prior SLE studies have often relied on cross sectional or time‐averaged SLEDAI summaries, which may obscure intra individual variability. Our findings suggest that GBTM applied to routine clinical data can identify distinct short‐term disease activity patterns that align with independently defined clinical status and renal‐related laboratory features. Despite the nonspecific nature of the urinary protein‐related measures used in this study, their longitudinal decline paralleled remission, supporting their pragmatic value in real world monitoring and illustrating how trajectory‐based assessment may complement, rather than replace, conventional disease activity indices.

Several limitations should be acknowledged. First, the trajectory groups identified in this study differed in baseline SLEDAI‐2K levels, indicating that baseline disease burden likely contributed substantially to subsequent trajectory membership. Therefore, our findings should not be interpreted as evidence that patients with the same initial disease activity will necessarily follow different short‐term courses. Rather, the present analysis is more appropriately viewed as identifying distinct short‐term disease activity patterns within a real world cohort. Second, although trajectory membership was associated with 6 month remission status, our study did not evaluate intermediate or long‐term outcomes such as damage accrual, flare recurrence, or mortality. Accordingly, the clinical implications of the identified trajectories should be interpreted cautiously in this context. Third, assessments were performed at only three predefined time points, namely, baseline, month 3, and month 6. Although this design captured early treatment response, it did not reflect the full spectrum of real‐world fluctuation, and denser and longer follow‐up will be required to evaluate trajectory stability and transitions [9]. Fourth, the dichotomous structure of SLEDAI‐2K items may reduce sensitivity to subtle changes over short time windows [28]. Fifth, urinary biomarkers were nonspecific, and medication exposure data were insufficient for inclusion as time‐varying covariates. Finally, our exploratory study did not compare the predictive accuracy of GBTM with that of cross sectional models. We, therefore, interpret the identified trajectories as providing complementary longitudinal insights rather than claiming superiority over traditional approaches. Multicenter prospective studies integrating continuous activity metrics, standardized therapy data, novel urinary biomarkers, and hard clinical outcomes are warranted to validate and extend these findings.

## 5. Conclusion

In conclusion, our study underscores the usefulness of GBTM in characterizing the heterogeneity of short‐term disease activity in SLE. By analyzing SLEDAI‐2K score trajectories over a 6 month period, we identified distinct patient subgroups with different disease activity patterns and 6 month clinical status. These findings may support a more nuanced understanding of disease dynamics and facilitate stratified clinical management in SLE.

## Author Contributions

Hongyan Hou and Ali Xiong wrote the manuscript. Rujia Chen, Renren Ouyang, and Wei Wei collected data from patient medical charts and also performed data entry. Ting Wang and Rujia Chen did the flow cytometry detection. Feng Wang and Shiji Wu analyzed the data and helped revise the manuscript. Shiji Wu is the guarantor.

## Funding

The study was supported by grants from the Hubei Provincial Health Commission (Grant WJ2023M010).

## Ethics Statement

This study was approved by the Ethics Committee of Tongji Hospital, Tongji Medical College, Huazhong University of Science and Technology (TJ‐IRB202308129).

## Conflicts of Interest

The authors declare no conflicts of interest.

## Supporting Information

Additional supporting information can be found online in the Supporting Information section.

## Supporting information


**Supporting Information 1** Table S1. It provides the detailed descriptors and weighted scores of SLEDAI‐2 K.

**Supporting Information 2** Figure S1. It provides the full gating strategy for flow cytometry analysis of lymphocyte subsets.

**Supporting Information 3** Table S2. It provides the fit statistics for the group based trajectory models of SLEDAI‐2K scores.

**Supporting Information 4** Table S3. It provides the clinical and laboratory characteristics of remission and nonremission patients in Class 2.

## Data Availability

The data that support the findings of this study are available from the corresponding author upon reasonable request.
