# Supplementary material for: Distinct Longitudinal Trajectories of SLEDAI‐2K Scores Predict Prognosis in Systemic Lupus Erythematosus Based on Group‐Based Trajectory Modeling
Source: J Immunol Res. 2026 Jun 30;2026:5322286. doi: 10.1155/jimr/5322286 (PMC13317468; doi:10.1155/jimr/5322286)
Supplement: Supplementary file 1 — Supporting Information 1 Table S1. It provides the detailed descriptors and weighted scores of SLEDAI‐2 K. [file JIMR-2026-5322286-s001.docx]

| Supplementary Table S1. Components and weighted scores of the SLEDAI-2K | | | |
| --- | --- | --- | --- |
| Domain | Descriptor | Definition / clinical meaning | Score |
| Neurologic | Seizure | Recent onset seizure attributable to active lupus, excluding metabolic, infectious, or drug-related causes | 8 |
|  | Psychosis | Lupus-related psychosis, excluding drug-induced or primary psychiatric causes | 8 |
|  | Organic brain syndrome | Altered mental function attributable to active SLE | 8 |
|  | Visual disturbance | Retinal or optic nerve involvement due to active lupus | 8 |
|  | Cranial nerve disorder | Cranial neuropathy attributable to SLE | 8 |
|  | Lupus headache | Severe persistent headache attributed to active lupus | 8 |
|  | Cerebrovascular accident | Stroke or focal neurologic event attributable to SLE | 8 |
|  | Vasculitis | Ulceration, gangrene, tender nodules, periungual infarction, splinter hemorrhages, or biopsy/angiographic evidence of vasculitis | 8 |
| Renal | Urinary casts | Heme-granular or red blood cell casts | 4 |
|  | Hematuria | > 5 red blood cells per high-power field, excluding other causes | 4 |
|  | Proteinuria | > 0.5 g/24 h or > 3+ if quantitation not performed | 4 |
|  | Pyuria | > 5 white blood cells per high-power field, excluding infection | 4 |
| Musculoskeletal | Arthritis | ≥ 2 joints with pain and signs of inflammation (tenderness, swelling, or effusion) | 4 |
|  | Myositis | Proximal muscle aching/weakness with elevated muscle enzymes or compatible investigations | 4 |
| Serosal | Pleurisy | Pleuritic chest pain with pleural rub/effusion attributable to SLE | 2 |
|  | Pericarditis | Pericardial pain, rub, effusion, or ECG/echo evidence attributable to SLE | 2 |
| Mucocutaneous | Rash | Inflammatory lupus rash | 2 |
|  | Alopecia | Active lupus-related abnormal diffuse or patchy hair loss | 2 |
|  | Mucosal ulcers | Oral or nasal ulcers attributable to active lupus | 2 |
| Immunologic / laboratory | Low complement | Decreased C3, C4, and/or CH50 attributable to active disease | 2 |
|  | Increased DNA binding | Elevated anti-dsDNA antibody binding above the normal range | 2 |
| Constitutional | Fever | Temperature > 38°C attributable to active lupus, excluding infection | 1 |
| Hematologic | Thrombocytopenia | Platelet count < 100 × 10⁹/L, excluding drug-related or other causes | 1 |
|  | Leukopenia | White blood cell count < 3 × 10⁹/L, excluding drug-related or other causes | 1 |

Note: **Abbreviation:** SLEDAI-2K, Systemic Lupus Erythematosus Disease Activity Index 2000.
