# Supplementary figures and images for: Distinct Longitudinal Trajectories of SLEDAI‐2K Scores Predict Prognosis in Systemic Lupus Erythematosus Based on Group‐Based Trajectory Modeling
Source: J Immunol Res. 2026 Jun 30;2026:5322286. doi: 10.1155/jimr/5322286 (PMC13317468; doi:10.1155/jimr/5322286)

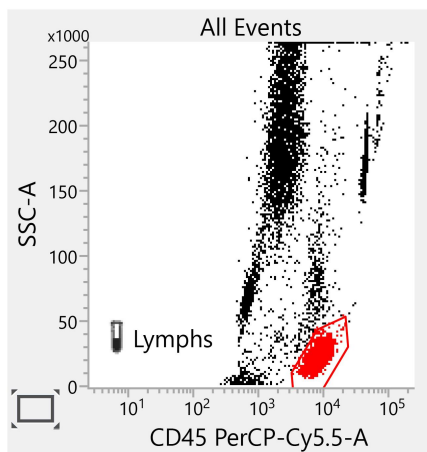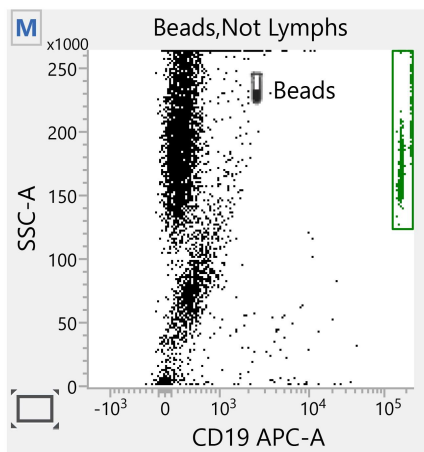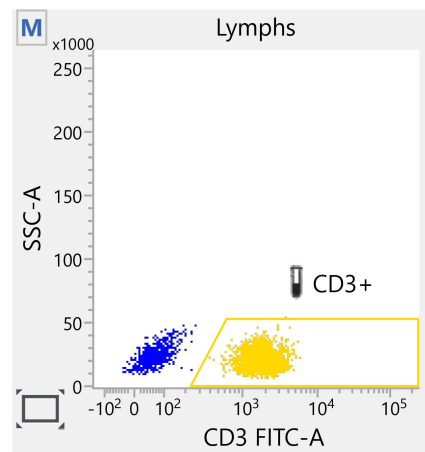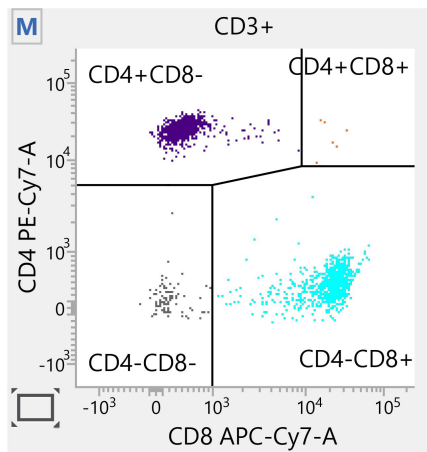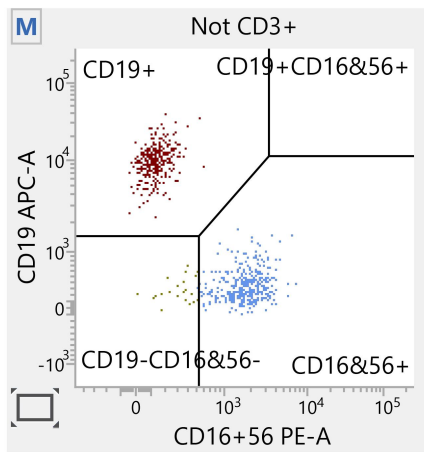

Supplement: Supplementary file 2 — Supporting Information 2 Figure S1. It provides the full gating strategy for flow cytometry analysis of lymphocyte subsets. [file JIMR-2026-5322286-s002.pdf]
