# Supplementary material for: Distinct Longitudinal Trajectories of SLEDAI‐2K Scores Predict Prognosis in Systemic Lupus Erythematosus Based on Group‐Based Trajectory Modeling
Source: J Immunol Res. 2026 Jun 30;2026:5322286. doi: 10.1155/jimr/5322286 (PMC13317468; doi:10.1155/jimr/5322286)
Supplement: Supplementary file 3 — Supporting Information 3 Table S2. It provides the fit statistics for the group based trajectory models of SLEDAI‐2K scores. [file JIMR-2026-5322286-s003.docx]

| Supplementary Table S2. Fit Statistics for the Development Trajectory of SLEDAI-2K Scores in Systemic Lupus Erythematosus | | | | | |
| --- | --- | --- | --- | --- | --- |
| Fit statistics | Number of classes | | | | |
|  | 1 | 2 | 3 | 4 | 5 |
| AIC | 1432.314928 | 1406.966836 | 1404.091271 | 1401.995171 | 1394.761018 |
| aBIC | 1446.352481 | 1438.551331 | 1453.222707 | 1468.673549 | 1471.967562 |
| CLass proportion | Class 1, 100% | Class 1, 48.35% | Class 1, 45.05% | Class 1, 30.77% | Class 1, 23.08% |
|  |  | Class 2, 51.65% | Class 2, 6.59% | Class 2, 7.69% | Class 2, 6.59% |
|  |  |  | Class 3, 48.35% | Class 3, 14.29% | Class 3, 41.76% |
|  |  |  |  | Class 4, 47.25% | Class 4, 25.27% |
|  |  |  |  |  | Class 5, 3.30% |
| APP |  | Class 1, 0.88 | Class 1, 0.89 | Class 1, 0.89 | Class 1, 0.83 |
|  |  | Class 2, 0.86 | Class 2, 0.84 | Class 2, 0.83 | Class 2, 0.81 |
|  |  |  | Class 3, 0.83 | Class 3, 0.84 | Class 3, 0.85 |
|  |  |  |  | Class 4, 0.82 | Class 4, 0.76 |
|  |  |  |  |  | Class 5, 0.99 |

Note: **Abbreviations:** AIC, Akaike information criterion; aBIC, adjusted Bayesian information criterion;

APP, average posterior probability. Lower AIC and aBIC values indicate better model fit.

Higher APP values indicate better classification accuracy, with values > 0.70 generally considered acceptable.

Each class should preferably include at least 5% of the total sample.
