# Supplementary material for: Distinct Longitudinal Trajectories of SLEDAI‐2K Scores Predict Prognosis in Systemic Lupus Erythematosus Based on Group‐Based Trajectory Modeling
Source: J Immunol Res. 2026 Jun 30;2026:5322286. doi: 10.1155/jimr/5322286 (PMC13317468; doi:10.1155/jimr/5322286)
Supplement: Supplementary file 4 — Supporting Information 4 Table S3. It provides the clinical and laboratory characteristics of remission and nonremission patients in Class 2. [file JIMR-2026-5322286-s004.docx]

| Supplementary Table S3. Clinical and laboratory characteristics of remission and non remission patients in Class 2 | | | |
| --- | --- | --- | --- |
| Parameters | Remission Group ( n = 25) | Non-Remission Group ( n = 22) | p value |
| Age , median [IQR], years | 37.000 [13.000-55.000] | 39.000 [19.000-52.000] | 0.365 |
| Sex |  |  |  |
| Male, n (%) | 5 (20.000) | 2 (9.091) | - |
| Female, n (%) | 20 (80.000) | 20 (90.909) | 0.295 |
| SLEDAI-2K score | 8.000 [8.000-12.000] | 9.000 [8.000-10.000] | 0.486 |
| Hematologic indicators |  |  |  |
| WBC (×109/L) | 4.530 [3.590-6.300] | 4.900 [3.860-6.960] | 0.509 |
| Lymphocytes (×109/L) | 0.870 [0.760-1.020] | 0.880 [0.620-1.330] | 1 |
| Neutrophilicgranulocytes (×109/L) | 3.170 [2.280-4.150] | 3.470 [2.620-4.470] | 0.639 |
| RBC (×1012/L) | 3.566±0.649 | 3.345±0.613 | 0.249 |
| Hemoglobin (g/L) | 101.760±20.080 | 99.136±21.188 | 0.672 |
| Platelet (×109/L) | 171.320±99.332 | 154.727±60.142 | 0.496 |
| Blood biochemistry indicators |  |  |  |
| Total protein (g/L) | 62.456±12.583 | 61.523±14.290 | 0.817 |
| GGT (U/L) | 26.000 [14.000-37.000] | 23.000 [13.000-32.000] | 0.594 |
| Cr (μmol/L) | 71.000 [50.000-103.000] | 80.000 [63.000-115.000] | 0.343 |
| Coagulation markers |  |  |  |
| PT (s) | 12.900 [12.000-13.500] | 12.800 [12.200-13.400] | 0.523 |
| FIB (g/L) | 3.500 [2.750-4.450] | 3.750 [2.780-4.670] | 0.66 |
| APTT (s) | 37.200 [34.200-42.000] | 35.700 [33.800-40.200] | 0.66 |
| TT (s) | 17.404±1.395 | 17.259±1.138 | 0.709 |
| D-dimer (μg/mL) | 1.780 [0.510-4.000] | 0.830 [0.520-2.620] | 0.271 |
| Urinary indicators |  |  |  |
| U-RBC (/μL) | 55.700 [25.100-125.900] | 63.600 [22.300-151.900] | 0.89 |
| U-WBC (/μL) | 26.200 [10.000-40.000] | 21.300 [16.900-41.200] | 0.423 |
| Urinary Casts (/μL) | 0.200 [0.000-0.400] | 0.300 [0.000-0.400] | 0.946 |
| 24h-UMA (mg/24 h) | 680.800 [204.200-1631.400] | 1164.900 [206.400-2824.200] | 0.337 |
| 24h-UMTP (mg/24 h) | 1041.600 [420.400-1979.200] | 1646.700 [501.100-4400.000] | 0.337 |
| U-MA (mg/L) | 545.900 [260.700-2039.300] | 1164.200 [593.800-3128.100] | 0.257 |
| U-MTP (mg/L) | 1020.000 [513.000-3077.000] | 1590.000 [998.000-3556.000] | 0.41 |
| Immune indicators |  |  |  |
| Total T cell (%) | 78.340 [74.560-83.150] | 80.200 [62.000-86.600] | 0.571 |
| Total B cells (%) | 11.420 [7.330-16.830] | 18.210 [7.180-30.000] | 0.284 |
| C3 (g/L) | 0.500 [0.250-0.600] | 0.450 [0.300-0.620] | 0.904 |
| C4 (g/L) | 0.100 [0.050-0.160] | 0.060 [0.050-0.150] | 0.566 |
| IgA (g/L) | 2.370±0.882 | 2.531±1.299 | 0.629 |
| IgG (g/L) | 14.100 [7.500-18.600] | 12.500 [8.200-21.900] | 0.59 |
| IgM (g/L) | 1.160 [0.710-1.740] | 0.820 [0.470-1.330] | 0.124 |

Note:Data are presented as n (%), median (interquartile range), or mean ± standard deviation (SD).

**WBC**, white blood cell count; **RBC**, red blood cell count; **GGT**, gamma glutamyl transferase; **Cr**, creatinine;

**PT**, prothrombin time; **FIB**, fibrinogen; **APTT**, activated partial thromboplastin time; **TT**, thrombin time;

**U-RBC**, urinary red blood cell count; **U-WBC**, urinary white blood cell count; **24h-UMA**, 24-hour urinary microalbumin;

**24h-UMTP**, 24-hour urinary micrototal protein; **U-MA**, urinary microalbumin; **U-MTP**, urinary micrototal protein;

**C3**, complement C3; **C4**, complement C4; **IgA**, immunoglobulin A; **IgG**, immunoglobulin G; **IgM**, immunoglobulin M.
